# Identification of Sex Pheromone Components for the Click Beetles *Melanotus piceatus* Blatchley and *Melanotus insipiens* (Say) (Coleoptera: Elateridae)

**DOI:** 10.1007/s10886-026-01702-w

**Published:** 2026-03-09

**Authors:** Livy Williams, Sean T. Halloran, Paul D. Baker, Frank E. Etzler, Jocelyn G. Millar

**Affiliations:** 1 USDA-ARS U.S. Vegetable Laboratory, 2700 Savannah Highway, Charleston, SC 29414 USA; 2 Departments of Entomology and Chemistry, University of California, Riverside, CA 92521 USA; 3 College of Agriculture, Health, and Natural Resources, Kentucky State University, 400 E. Main St., Frankfort, KY 40601 USA; 4 Montana Department of Agriculture, 302 N. Roberts St., Helena, MT 59714 USA

**Keywords:** Elateridae, Click beetles, Wireworms, Melanotus piceatus, Melanotus insipiens, Sex pheromone, Attractant

## Abstract

Little is known about the semiochemicals that mediate the reproductive behavior of click beetles, the Elateridae. Research over the past two decades has begun to fill this gap, with: (1) the discovery of sex attractants for a number of pest and non-pest species, and (2) subsequent studies toward development of semiochemically-based pest management approaches and conservation initiatives for pests and non-pests, respectively. We used chemical, electrophysiological, and behavioral studies to identify, synthesize, and field test female-produced sex attractant pheromones of two North American elaterid species, *Melanotus piceatus and M. insipiens*. We identified two possible pheromone components for each species using coupled gas chromatography-mass spectrometry analyses of extracts of ovipositors of females: (2*Z*,6*E*)-farnesyl acetate and (2*E*,6*E*)-farnesyl acetate for *M. piceatus*, and decyl octanoate and decyl butanoate for *M. insipiens*. Subsequent coupled gas chromatography-electroantennogram detection analyses indicated that antennae of males were responsive to only one of the two compounds for each species: (2*Z*,6*E*)-farnesyl acetate for *M. piceatus*, and decyl butanoate for *M. insipiens*. In field trials, (2*Z*,6*E*)-farnesyl acetate and decyl butanoate as single components attracted males of *M. piceatus* and *M. insipiens*, respectively. In both species, most male beetle flight activity occurred from May through June. Identification of the sex pheromones of these species will provide useful tools to study their biology, while further expanding our knowledge of the types of compounds to be expected in the sex pheromones of related species.

## Introduction

To date, the North American click beetles (Coleoptera: Elateridae) are represented by about 1,000 described species (Johnson 2002). Various members feed on plant and animal tissues as well as detritus, and this biomass recycling makes them valuable components of natural ecosystems. Some herbivorous species are important pests of a wide array of crops (reviewed in Rashed and van Herk 2024; Traugott et al. 2015), whereas others are cryptic and infrequently encountered, but are nevertheless important members of ecosystems. Regardless of their pest status, little is known about the biology and natural history of most species. This is in part due to the concealed habitats of immature stages (i.e., subterranean, decaying wood, litter), the cryptic appearance of adults of most species (Douglas et al. 2024), and the multiyear life cycles of many species. These factors render studies of elaterid biology expensive, logistically challenging, labor-intensive, and time-consuming.

The reproductive biology of click beetles has been a growing focus of research over the past ~ 20 years, and we have learned that females of many species produce sex attractant pheromones to attract males for mating. For example, pheromones and sex attractants have been identified for several European and Asian species (e.g., Tamaki et al. 1990; Tolasch et al. 2007; reviewed in Tóth 2013). The first pheromones for North American species were reported relatively recently, in 2018, for two *Cardiophorus* Eschscholtz species (Serrano et al. 2018). Since then, pheromones or sex attractants have been discovered for several pest species, including *Melanotus communis* (Gyllenhal) (Williams et al. 2019) and M. *verberans* (LeConte) (Williams et al. 2025), *Limonius californicus* (Mannerheim), *L. canus* LeConte (Gries et al. 2021), L. *agonus* (Say), and *L. infuscatus* (Motschulsky) (van Herk et al. 2021), and *Selatosomus destructor* (Brown) (Gries et al. 2022). Pheromones or sex attractants have also been reported for North American species which are not known to be pests, including *Parallelostethus attenuatus* (Say) (Millar et al. 2022), *Idolus* Desbrochers des Loges and *Dalopius* Eschscholtz species (Serrano et al. 2022), *Elater abruptus* Say, *M. ignobilis* Melsheimer, *Agriotes insanus* Candèze, *Gambrinus griseus* (Palisot de Beauvois), *G. plebejus* (Say), and *G. rudis* (Brown) (Millar et al. 2024), and most recently, *Dalopius maritimus* Brown and *D. asellus* Brown (van Herk et al. 2025). In total, these data suggest that sex pheromones are important components of the reproductive biology of both pest and non-pest elaterids.

As part of a long-term project to elucidate the semiochemistry of the Elateridae, the goal of the present study was to identify, synthesize, and evaluate sex pheromone components of two *Melanotus* species, *M. piceatus* Blatchley and *M. insipiens* (Say). *Melanotus piceatus* is a moderate-sized beetle (11–12 mm length), elongate with dark red-brown to black coloring, and occurs in the southeastern U.S. (Mathison 2021; Quate and Thompson 1967). *Melanotus insipiens* is small (5–6.5 mm length), with a light to dark red-brown coloring, and is found in the eastern U.S. from New Jersey to Florida and west to Texas (Mathison 2021; Quate and Thompson 1967). Little is known about the life history of either species, but immature stages probably inhabit the soil for months or years before pupating, similar to other *Melanotus* species (Douglas et al. 2024). Adult beetles are active above-ground in the spring and summer, at which time they mate and females lay eggs, probably in or on the soil. *Melanotus insipiens* has been recorded as a pest of alsike clover and wheat in Georgia, U.S.A. (Fattig 1951). Identification of the sex pheromones of these two species will promote studies of their basic biology, including taxonomy, geographic range, and natural history, while also improving our knowledge of the types and use of sex pheromones within the family.

## Methods and Materials

### Collection and Maintenance of Beetles

Live *Melanotus piceatus* and *M. insipiens* adults were collected from agricultural fields and adjacent wooded areas at the USDA-ARS U.S. Vegetable Laboratory (USVL) in Charleston, SC, USA, (32°48′02.06″N 80°03′40.35″W, elev. 4 m for *M. piceatus*; 30 May 2022, UV light), Clemson University Pee Dee Research and Extension Center (CUPDREC) in Florence, SC, USA, (34°17′40.60″N 79°43′35.00″W, elev. 35 m for *M. insipiens*; 29 May and 3 June 2023, beating vegetation), and a residential neighborhood in Florence, SC, USA, (34°11′09.20″N 79°47′24.00″W, elev. 43 m for *M. piceatus*; 1 June 2023, incandescent porch light). Mating status, age, and other life history factors of the beetles were not known. Additionally, in 2024, male beetles of both species were live-trapped for coupled gas chromatography-electroantennogram detection (GC-EAD) assays using funnel traps (McQuate 2014) baited with (2*Z*,6*E*)-farnesyl acetate for *M. piceatus* and a blend of decyl octanoate: decyl butanoate (10:3.3) for *M. insipiens* (see below). After collection, beetles were held individually in ventilated transparent plastic vials streaked with honey and containing a piece of paper towel moistened with distilled water. Insects were held (22 °C ± 1, 50% r.h., L: D 16:8) for 1 to 7 d prior to overnight shipment to the University of California, Riverside, Entomology Quarantine Facility (USDA-APHIS permit P526P-20–00853) for extractions, and chemical and electrophysiological analyses. All *Melanotus* species were identified by comparison with reference specimens of known identity, supported with genital morphology and taxonomic keys (Mathison 2021; Quate and Thompson 1967). Voucher specimens are deposited in the Clemson University Arthropod Collection, Clemson, SC, and the National Museum of Natural History through the USDA-ARS Systematic Entomology Laboratory, Washington, D.C.

### Extraction and Analysis of Potential Sex Pheromone Components

Female beetles were cold anesthetized and the ovipositor and associated glandular structures were pulled out of the abdomen tip with forceps. Ovipositors were extracted individually overnight in 0.1 ml CH_2_Cl_2_, then the extracts were transferred to screwcap autosampler vials fitted with fused 0.2 ml inserts. Extracts were analyzed by coupled gas chromatography-mass spectrometry (GC-MS) using a DB-5 column in EI mode at 70 eV, as previously described (Williams et al. 2025). Further GC-MS analyses were made with a DB-17 column (30 m x 0.25 mm ID x 0.25 µ film thickness, J&W Scientific) on an HP-5890 instrument coupled to a 5970B mass selective detector. The GC was programmed from 40 °C for 1 min, increased 10 °C/min to 280 °C, hold for 20 min, with 1 µl injections made in splitless mode. Compounds were tentatively identified by interpretation of their mass spectra. Identifications were confirmed by matching retention times and mass spectra with those of authentic standards. Linear retention indices were calculated based on a standard blend of C_8_-C_40_ alkanes.

### Coupled Gas Chromatography-Electroantennogram Detection (GC-EAD) Analyses of Potential Sex Pheromone Components

Antennae of live-trapped males of both species were challenged with ovipositor extracts and solutions of authentic standards in GC-EAD analyses. Before male beetles were removed from the quarantine facility to the GC-EAD laboratory, the legs were removed, and their genitalia were glued shut with Super Glue^®^, as required by the quarantine permit. An antenna was removed from the head with forceps, and mounted between saline-filled capillary glass electrodes, with a gold wire providing connection to a custom-built EAD amplifier. The GC conditions were as described by Williams et al. (2025). The GC and EAD detector outputs were recorded simultaneously and displayed with Peak Simple^®^ software (SRI Instruments, Torrance CA, USA).

### Chemicals

(2*E*,6*E*)-Farnesyl acetate was purchased from Aldrich Chemical Co. (Milwaukee WI) and (2*Z*,6*E*)-farnesyl acetate, decyl octanoate and decyl butyrate were synthesized as described below.

Reactions sensitive to air and/or water were carried out in oven-dried glassware under Ar. Unless otherwise specified, solutions were concentrated by rotary evaporation, and dried over anhydrous Na_2_SO_4_. Compounds were purified by flash chromatography on 230–400 mesh silica gel. Mass spectra were taken with a Hewlett-Packard 6890 GC interfaced to an HP 5973 mass selective detector. The GC was equipped with an intermediate polarity DB-17 column (30 m x 0.25 mm ID x 0.25 µ film thickness, J&W Scientific), and was programmed from 40 °C for 1 min, then increased 10 °C/min to 280 °C, hold 10 min.

### Synthesis of (2*Z*,6*E*)-Farnesyl Acetate

(2*E*,6*E*)-Farnesol (1.11 g, 5 mmol, Aldrich Chemical, Milwaukee WI, USA) was added in one portion to a slurry of powdered pyridinium chlorochromate (1.62 g, 7.5 mmol) in 75 ml CH_2_Cl_2_ and the mixture was stirred 2 h. Ether (100 ml) was added, and the mixture was stirred 10 min, then filtered through a pad of silica gel, rinsing well with ether. The filtrate was concentrated to a yellow-green oil, yielding a 78:17 mixture of the (2*E*) and (2*Z*) isomers of the corresponding aldehydes, substantially different than the literature report (~ 36% (2*Z*), Mitachi et al. 2015). To increase the proportion of the (2*Z*) isomer, the crude product was taken up in 25 ml ether, *p*-toluenesulphonic acid (0.3 g, 1.6 mmol) was added, and the mixture was stirred at room temp overnight, resulting in an improved 57:38 ratio of the (2*E*) and (2*Z*) isomers. The solution was diluted with hexane, washed with saturated aq. NaHCO_3_ and brine, dried, and concentrated. The residue was purified by flash column chromatography on silica gel (5 cm diam x 22 cm bed height), eluting with 4% EtOAc in hexane (1.5 L), yielding a purified fraction of (2*Z*,6*E*)-farnesal (190 mg), which eluted first. This was taken up in 5 ml MeOH, the solution was cooled in an ice bath, NaBH_4_ (0.04 g, 1.05 mmol) was added, the mixture was stirred 30 min, then quenched by addition of saturated aq. NH_4_Cl, and most of the MeOH was removed by rotary evaporation. The residue was partitioned between hexane and water, and the hexane solution was dried and concentrated, yielding (2*Z*,6*E*)-farnesol. This was taken up in 5 ml CH_2_Cl_2_, pyridine (0.16 ml, 2 mmol) and dimethylaminopyridine (~ 20 mg) were added, and the solution was cooled in an ice bath while adding 0.157 g (2 mmol) acetyl chloride in 1 ml CH_2_Cl_2_ over 30 min with a syringe pump. The mixture was warmed to room temperature overnight, EtOH (0.1 ml) was added to quench any remaining acetyl chloride, and after stirring 1 h, the CH_2_Cl_2_ was removed by rotary evaporation and the residue was partitioned between water and hexane. The hexane solution was washed with saturated aq. NaHCO_3_ and brine, dried, and concentrated. The crude farnesyl acetate was purified by vacuum flash chromatography, eluting with 5% EtOAc in hexane.

A mixed sample of the farnesyl acetate isomers was prepared in similar fashion from a sample of farnesol (Aldrich Chemical) containing all four isomers.

### Synthesis of Decyl Octanoate

Octanoyl chloride (3.26 g, 20 mmol) was added dropwise to a stirred solution of decanol (3.16 g, 20 mmol), pyridine (1.58 g, 20 mmol), and ~ 25 mg dimethylaminopyridine catalyst in 100 ml CH_2_Cl_2_ cooled in an ice bath. After the addition was complete, the mixture was warmed to room temperature, stirred 3 h, then poured into water and shaken vigorously. After separating the layers, the organic layer was washed sequentially with 1 M HCl, saturated aq. NaHCO_3_, and brine, then dried. After concentration, the residue was purified by vacuum flash chromatography, eluting with 5% ethyl acetate in hexane, followed by Kugelrohr distillation (bp ~ 105 °C, 0.05 mm Hg), yielding the ester as a colorless oil (4.15 g, 73%).

Decyl butanoate was synthesized in a similar fashion by substituting butanoyl chloride for octanoyl chloride.

### Field Bioassays of Synthetic Pheromone Candidates

Bioassays in 2023 and 2024 were set up using transects of traps (Vernon Pitfall Traps^®^, ca. 17 × 14 cm, (VPT) (van Herk et al. 2018) constructed of black polypropylene and deployed 15 m apart. VPTs were positioned 1 m above the ground on black plastic stakes (Schoeppner et al. 2023). In all trials, trap lures consisted of 9 mm grey rubber septa (West Pharmaceutical Services, Lititz, PA, USA) loaded with test compounds in 0.1 ml hexane, with 2 mg of the major component and, in some trials, variable amounts of a possible minor component. Solvent control lures were loaded with 0.1 ml of hexane. Lures were suspended with a paper clip from the center of the inside of the trap lid. Each VPT held a 250 ml polypropylene sample cup (Falcon 354015, Corning Life Sciences, Corning, NY) containing ca. 12 ml of a 1:1 mixture of water and propylene glycol (Prestone LowTox^®^ Antifreeze/Coolant; Prestone Products Corp., Lake Forest, IL, USA) to serve as a killing agent/preservative. Click beetles were removed from traps weekly and lures were replaced and treatment locations in each transect were re-randomized every 3 wk.

Bioassays in 2023 were conducted using putative *M. piceatus* attractants at three locations in South Carolina: USVL (32°48′02.55″N 80°03′37.35″W, elev. 4 m), CUPDREC (34°17′40.60″N 79°43′35.00″W, elev. 35 m), and a commercial tree farm (CTF) in Liberty, SC (34°46′04.80″N 82°40′21.40″W, elev. 254 m). Details of field bioassays for 2023 and 2024 are given in Table 1. Field bioassays (= trap transects) were conducted at three sites at USVL, and one site each at CUPDREC and CTF for 22 wk (2 April – 5 September 2023). One of the USVL transects was at the edge of a woodlot adjacent to a block of organic fallow fields (6 ha), and the other transect was established along an unpaved farm road bisecting a woodlot 1.2 km distant from the first transect. The transect at CUPDREC was established on the edge between a woodlot and a fallow field, and the transect at CTF was established between nursery plots of ornamental trees. In 2023, two test lures were prepared; (1) a synthetic pheromone blend approximating the ratio determined from analysis of *M. piceatus* ovipositor extracts ((2*Z*,6*E*-farnesyl acetate: (2*E*,6*E*)-farnesyl acetate, mean ratio from two specimens 100:1.4)), and (2) the major component (2*Z*,6*E*)-farnesyl acetate alone.

**Table 1:** Study sites, compounds tested, collection dates and other information for the field bioassays for *Melanotus piceatus* and *M. insipiens* in South Carolina, 2023–2024

Species	Year/Experiment	Site/no. trials^1^	Study dates	Compounds^2, 3, 4^	No. replicates
*M. piceatus*	2023/1 2024/1	USVL/2, CUPDREC/1, CTF/1 USVL/3, CUPDREC/2, SVLE/1	2 April-5 September 16 April – 1 August	(2*Z*,6*E*)-FA: (2*E*,6*E*)-FA; 100:1.4) and (2*Z*,6*E*)-FA (2*Z*,6*E*)-FA	5 5
*M. insipiens*	2024/1 2024/2	USVL/3, CUPDREC/1 USVL/3, CUPDREC/2	17 April-28 May 28 May-11 July	DO: DB; 10:0, 10:0.1, 10:0.33, 10:1, 10:3.3 DO: DB; 10:3.3, 10:10, 0:10	10 6

^1^USVL = Charleston, SC (32°48′02.55″N 80°03′37.35″W, elev. 4 m)

CUPDREC = Florence, SC (34°17′40.60″N 79°43′35.00″W, elev. 35 m)

CTF = Liberty, SC (34°46′04.80″N 82°40′21.40″W, elev. 254 m)

SVLE = Summerville, SC (32°58’38.92”N 80°09’40.60”W, elev. 25 m)

^2^(2*Z*,6*E*)-FA: (2*E*,6*E*)-FA = (2*Z*,6*E*)-farnesyl acetate: (2*E*,6*E*)-farnesyl acetate; (2*Z*,6*E*)-FA = (2*Z*,6*E*)-farnesyl acetate

^3^DO: DB = Decyl octanoate: decyl butanoate

^4^Controls treated with hexane only were included in each trial

In 2024, field bioassays for *M. piceatus* were conducted at USVL (three transects), Summerville (SVLE) (one transect), and CUPDREC (two transects). The SVLE site was a suburban woodlot (ca. 22 km northwest of USVL, 32°58’38.92”N 80°09’40.60”W, elev. 25 m) representative of the Lower Coastal Plain plant community (Porcher and Rayner 2001). At USVL, the two transects from 2023 were used, and an additional transect was set up on a two-track road bisecting woods. All transects at USVL were within 1.2 km of each other. At CUPDREC, the transect from 2023 was used, and an additional transect was established between a woodlot and a fallow field 1.6 km away from the first transect. Trapping was conducted with the one component lure for *M. piceatus* [(2*Z*,6*E*)-farnesyl acetate] for 15 wk, from 16 April – 1 August.

Field bioassays for *M. insipiens* were conducted only in 2024 (Table 1). We used a semi-log scale of varying ratios of the two compounds found in ovipositor extracts (decyl octanoate and decyl butanoate; 10:0, 10:0.1, 10:0.33, 10:1, 10:3.3) during the first 6 wk of the study (17 April – 28 May). This study was conducted at four sites (the three transects at USVL described above for *M. piceatus* in 2024, and one transect at CUPDREC described above for *M. piceatus* in 2023). Based on the results, a second study was set up for another 6 wk, from 28 May – 11 July, with a different set of treatments (10:3.3, 10:10, 0:10 decyl octanoate: decyl butanoate). This study was conducted at the same five transects at USVL and CUPDREC used for *M. piceatus* in 2024.

### Statistical Analysis

Replicates were defined by the transect-weekly sample date combinations. For instance, in the 2023 *M. piceatus* field study there were 5 transect-sample date combinations where beetles were captured (16 May to 20 June), thus 5 replicates for analysis (Table 1). In 2023, only eight beetles were captured in the treatment with the 100:1.4 ratio of (2*Z*,6*E*)-farnesyl acetate: (2*E*,6*E*)-farnesyl acetate, so these data were excluded from analyses. In the 2024 *M. piceatus* field study there also were 5 transect-sample date combinations where beetles were captured (16 May to 6 June and 20 to 27 June), thus 5 replicates for analysis. For both species, the numbers of male beetles captured in each treatment were summed for each transect-sample date combination, and were transformed [log_10_(y + 1.5)] to mitigate non-normality (Sokal and Rohlf 1995). Transformed data for the treatment with the major component alone, (2*Z*,6*E*)-farnesyl acetate, were subjected to a one-tailed Welch’s t-test vs. the hexane controls. Likewise, for *M. piceatus* in 2024, data with the major component alone vs. hexane controls were subjected to a one-tailed Welch’s t-test. Seasonal phenology for *M. piceatus* is presented as the number of beetles captured with the main component, (2*Z*,6*E*)-farnesyl acetate, for each sample date. For *M. insipiens* (only studied in 2024), separate analyses were conducted for each trial. In the first field study there were 10 transect-sample date combinations where beetles were captured (25 April to 30 May), thus 10 replicates for analysis (Table 1). In the second field study there were several instances where only one or two beetles were captured in a replicate so those replicates were not included in analyses; this resulted in 6 transect-sample date combinations where beetles were captured (30 May to 27 June), thus 6 replicates for analysis. For each trial, transformed data for treatments and hexane controls were subjected to single-factor ANOVA followed by Dunnett’s test to compare each treatment to a selected control, i.e., the natural ratio of 10:3.3 for decyl octanoate: decyl butanoate. Untransformed means *±* SE across all sample dates are presented in results. Seasonal phenology for *M. insipiens* is presented as the number of beetles captured with the natural blend, 10:3.3 decyl octanoate: decyl butanoate, for each sample date.

## Results

### Identification of the Pheromone Component of *M. piceatus*

**Fig. 1:**
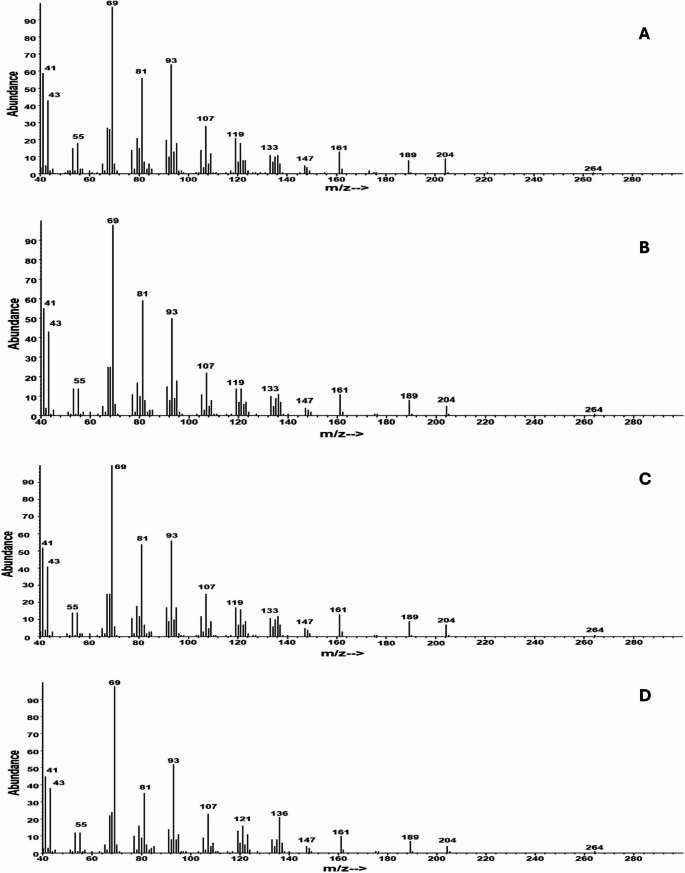
EI mass spectra of (**A**) (2*Z*,6*Z*)-farnesyl acetate, (**B**) (2*E*,6*Z*)-farnesyl acetate, (**C**) (2*Z*,6*E*)-farnesyl acetate, and (**D**) (2*E*,6*E*)-farnesyl acetate found in an ovipositor extract of a *Melanotus piceatus* female

The mass spectrum of the single major component looked like that of a sesquiterpene hydrocarbon, with a significant ion at *m/z* 204, and typical sesquiterpene-like ions at e.g., *m/z* 189, 161, 133, 119, 93, 79, and 69, and one of the best database matches was to α-farnesene. However, the retention time was markedly longer than that of α-farnesene, and crucially, there was a small molecular ion at *m/z* 264, and a small ion at *m/z* 60 (CH_3_COOH) corresponding to acetic acid, suggesting that the compound was the acetate ester of a sesquiterpene alcohol, possibly a farnesyl acetate (Fig. 1). Although the retention time was slightly earlier and the mass spectrum did not quite match that of a standard of (2*E*,6*E*)-farnesyl acetate (Fig. 1d), they did match those of one of the four peaks in a farnesyl acetate standard containing all four isomers, in particular, the isomer which eluted third on a nonpolar DB-5 GC column. Given that the order of elution of the four farnesol isomers on the same stationary phase is (2*Z*,6*Z*), (2*E*,6*Z*), (2*Z*,6*E*), and (2*E*,6*E*) (Adams 2007), we reasoned that the unknown might be (2*Z*,6*E*)-farnesyl acetate. Synthesis of a standard showed this to be correct, with the mass spectrum and retention indices on both a DB-5 column and a medium polarity DB-17 column (Table 2) matching those of the insect-produced compound. The retention indices of the (2*E*,6*Z*)- and (2*Z*,6*E*)-isomers were indistinguishable on the DB-17 column, but were slightly different on the DB-5 column (Table 2). In addition, differences in the ion ratios of the four isomers were helpful in distinguishing the four isomers (Fig. 1).

**Table 2:** Linear retention indices (RI) of farnesyl acetate isomers, decyl butanoate, and decyl octanoate on DB-5 and DB-17 GC columns

Compound	RI on DB-5	RI on DB-17
(2*Z*,6*Z*)-farnesyl acetate	1776	1959
(2*E*,6*Z*)-farnesyl acetate	1804	1997
(2*Z*,6*E*)-farnesyl acetate	1808	1997
(2*E*,6*E*)-farnesyl acetate	1831	2027
Decyl butanoate	1584	1684
Decyl octanoate	1971	2075

The mass spectrum and retention indices of a possible minor component, eluting shortly after the major component on both columns, matched those of (2*E*,6*E*)-farnesyl acetate, confirming its identity (Fig. 1d; Table 2).

**Fig. 2:**
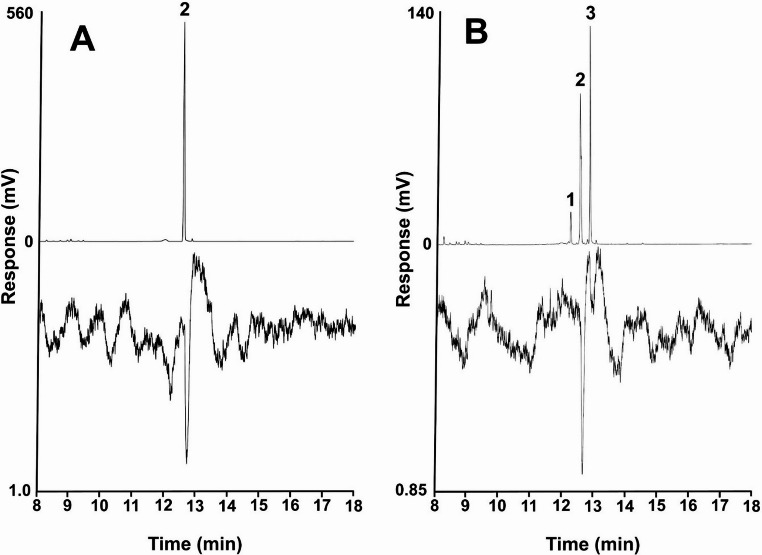
Representative coupled gas chromatography-electroantennograms of (**A**) an ovipositor extract of a *Melanotus piceatus* female and (**B**) a synthetic blend of the four farnesyl acetate isomers. Upper trace is the gas chromatogram (DB-5 column), lower trace is the antennal response of a male beetle. Compound identifications are: (1) (2*Z*,6*Z*)-farnesyl acetate, (2) (2*Z*,6*E*) and (2*E*,6*Z*)-farnesyl acetate, and (3) (2*E*,6*E*)-farnesyl acetate

GC-EAD analysis of an ovipositor extract showed only a single antennal response at the retention time of the major component, (2*Z*,6*E*)-farnesyl acetate (Fig. 2a), and GC-EAD analysis of the mixed standard of all four farnesyl acetate isomers likewise showed one major response at the retention time of (2*Z*,6*E*)-farnesyl acetate (Fig. 2b), suggesting that the pheromone consisted of that compound as a single component.

### Identification of Potential Pheromone Components of *M. insipiens*

**Fig. 3:**
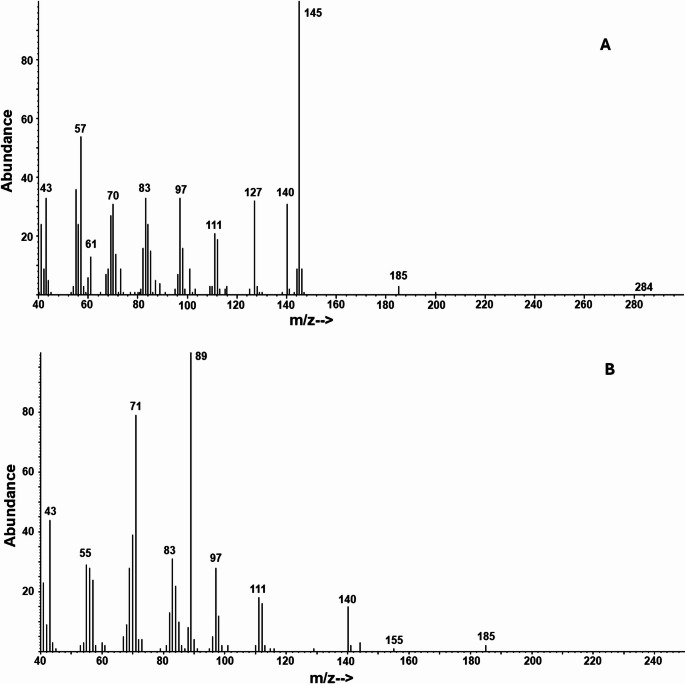
EI mass spectra of the two compounds identified in an ovipositor extract of a *Melanotus insipiens* female. Compound identifications: (**A**) decyl octanoate and (**B**) decyl butanoate

**Fig. 4:**
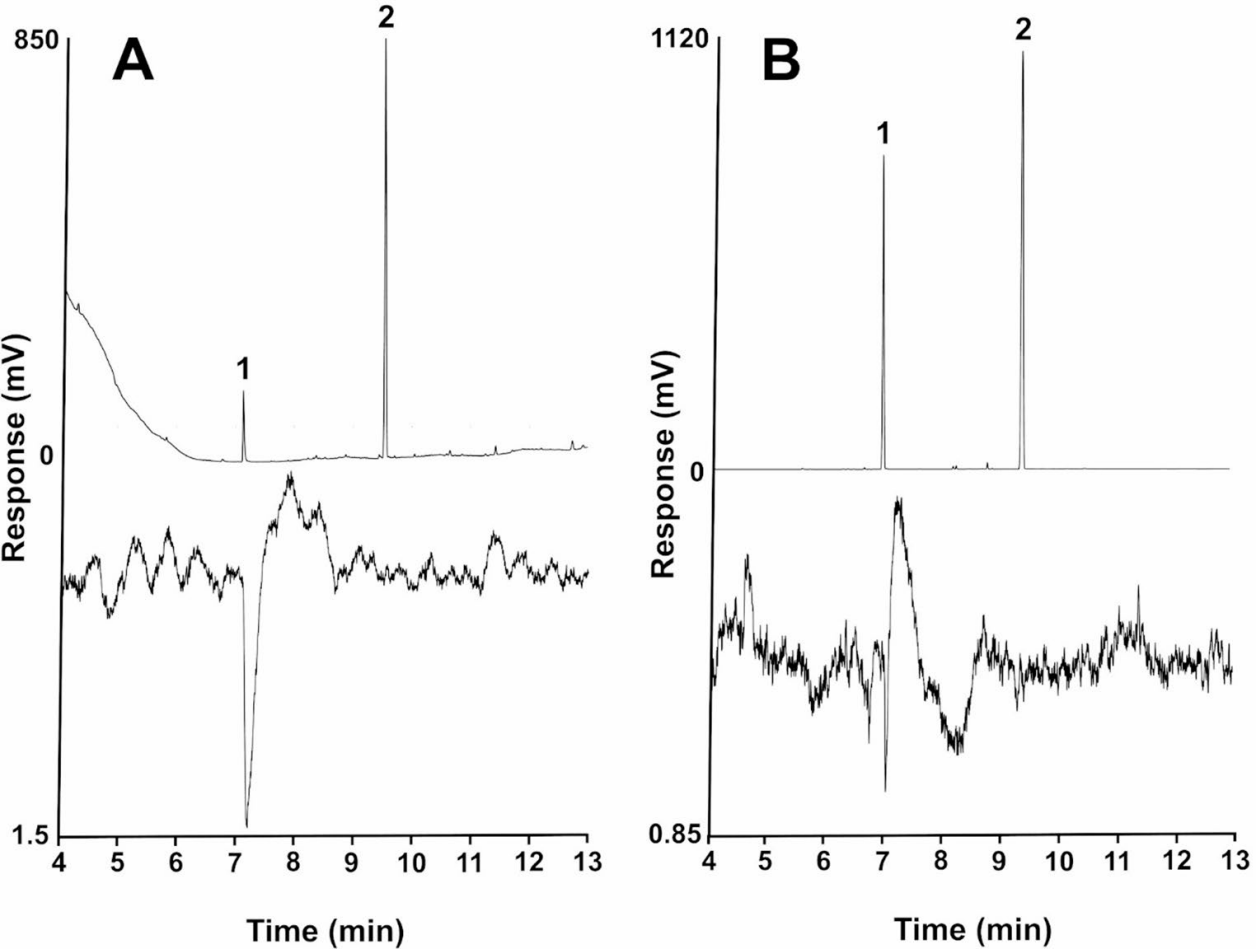
Representative coupled gas chromatography-electroantennograms of (**A**) an ovipositor extract of a *Melanotus insipiens* female and (**B**) a synthetic blend of the two compounds. Upper trace is the gas chromatogram (DB-5 column), lower trace is the antennal response of a male beetle. Compound identifications are: (1) decyl butanoate and (2) decyl octanoate

GC-MS analyses of hexane extracts of ovipositors of female *M. insipiens* showed essentially two peaks, in a ratio of 6.1:100 (*n* = 3). The major, later-eluting peak was tentatively identified as decyl octanoate by its trace molecular ion at *m/z* 284 (C_18_H_36_O_2_), a pair of ions separated by 18 amu at *m/z* 127 (C_8_H_15_O) and *m/z* 145 (C_8_H_17_O_2_, base peak) from the acid portion of the ester, and a prominent ion at *m/z* 140 (C_10_H_20_) from the alcohol portion of the ester (Fig. 3a). Its spectrum also was a reasonable match for the NIST 14 database spectrum for this compound, and the identification was confirmed by matching its retention indices (Table 2) and mass spectrum with those of an authentic standard. The minor, earlier eluting peak did not show a molecular ion, but it had a significant *m/z* 140 ion (C_10_H_20_), suggesting a decyl ester, along with a pair of ions separated by 18 mass units at *m/z* 71 (C_4_H_7_O) and the base peak at *m/z* 89 (C_4_H_9_O_2_), characteristic of a butanoate ester (Fig. 3b). The spectrum was a reasonable match for the database spectrum for decyl butanoate, and the identification was confirmed by matching its mass spectrum and retention indices (Table 2) with those of a synthetic standard. Unexpectedly, GC-EAD analysis of an ovipositor extract showed only a single antennal response to the minor component decyl butanoate (Fig. 4a), and GC-EAD analysis of the mixed standard of decyl octanoate and decyl butanoate likewise showed only a single response at the retention time of decyl butanoate (Fig. 4b).

### Field Bioassays of Synthetic Pheromone Candidates

#### *Melanotus piceatus*

**Fig. 5:**
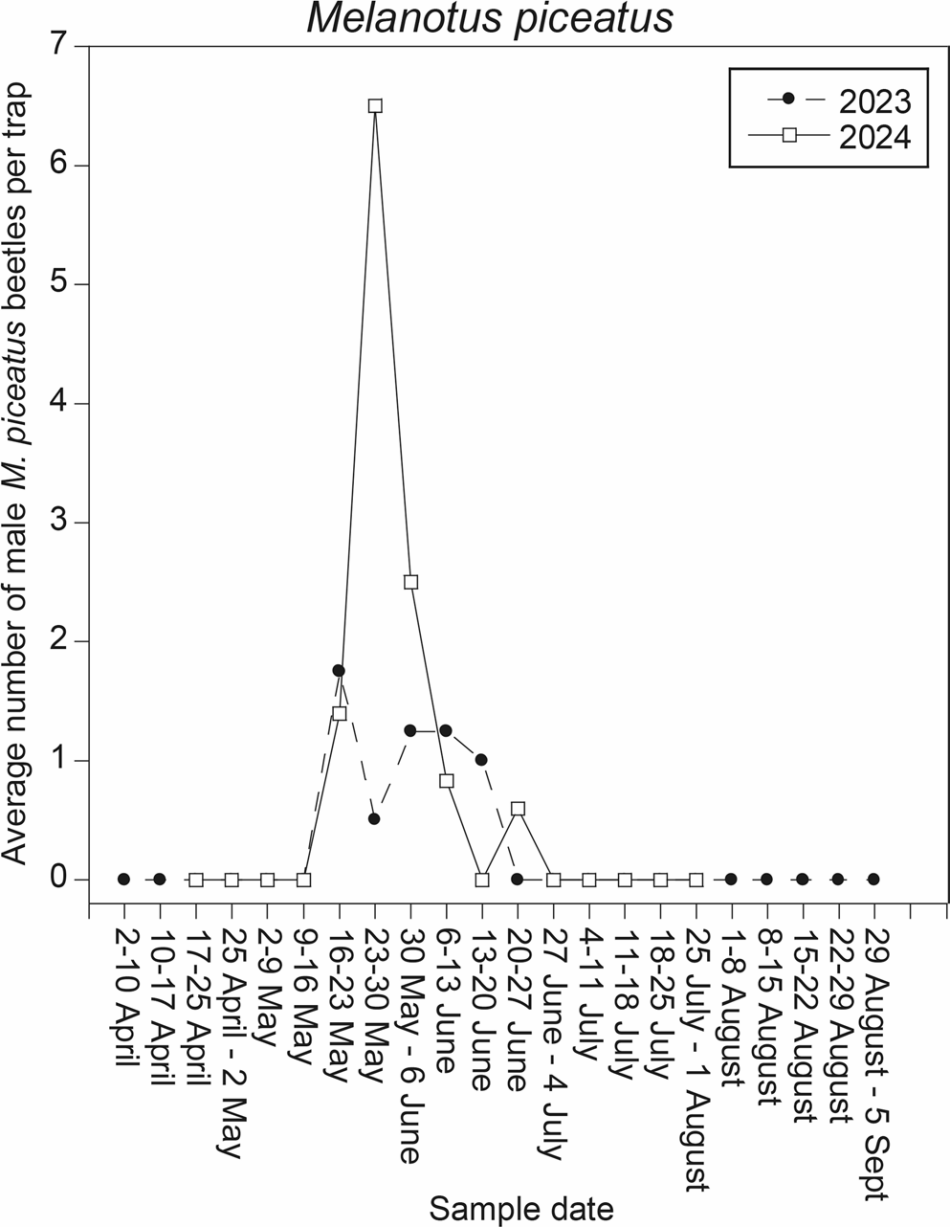
Seasonal phenology of male *Melanotus piceatus* captures with (2*Z*,6*E*)-farnesyl acetate lures, 2023 and 2024

In 2023, a total of 31 males were captured in traps baited with treatment lures. Only eight beetles were captured in the two-component blend of (2*Z*,6*E*)-farnesyl acetate: (2*E*,6*E*)-farnesyl acetate (mean ratio from two specimens 100:1.4), so these data were not included in further analyses. Twenty-three beetles were captured with the single component (2*Z*,6*E*)-farnesyl acetate, and no beetles were captured in control traps (*t* = 9.30, df = 4, *P* < 0.001). Beetles were captured for 5 wk (16 May to 20 June) during the 22 wk trial in 2023 (Fig. 5). Based on these results, and the fact that in GC-EAD analyses only the (2*Z*,6*E*)-isomer had elicited antennal responses, subsequent field trials in 2024 tested (2*Z*,6*E*)-farnesyl acetate as a single component.

In 2024, 56 male beetles were captured in traps baited with (2*Z*,6*E*)-farnesyl acetate, with no beetles in control traps (*t* = 5.90, df = 4, *P* = 0.002). Beetles were captured from 16 May to 13 June and from 20 to 27 June in 2024 (Fig. 5).

#### *Melanotus insipiens*

**Fig. 6:**
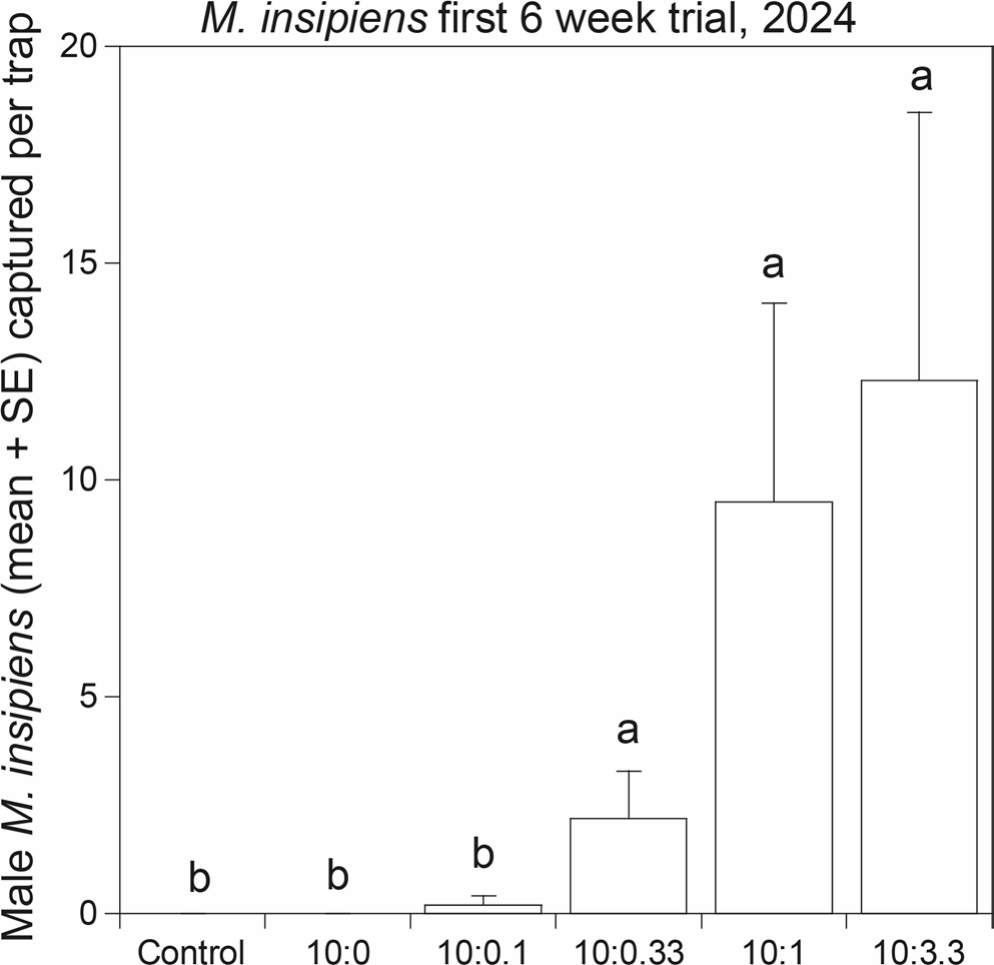
First 6 wk field bioassay (16 April – 28 May 2024) testing attraction of male *Melanotus insipiens* to the blend of decyl octanoate: decyl butanoate (10:3.3) found in an ovipositor extract (column at the right) versus other blends, on a semi-log scale. Columns represent the mean number of male beetles caught per trap for each treatment. Columns surmounted with the same letters are not significantly different (ANOVA, F = 4.25, df = 5,59, *P* = 0.002, *n* = 242, followed by Dunnett’s Test comparing the 10:3.3 blend to each of the other treatments, *P* < 0.05)

In the first trial in 2024, testing five blends (10:0, 10:0.1, 10:0.33, 10:1, 10:3.3; decyl octanoate: decyl butanoate), a total of 242 males were captured in traps baited with the various treatments, with 0 male beetles in control traps (*F* = 4.26, df = 5,59, *P* = 0.002) (Fig. 6). One female *M. insipiens* was captured in a control trap (first CUPDREC transect, 20–28 May). No beetles were captured in traps baited with decyl octanoate as the sole component, and trap captures appeared to increase as the proportion of decyl butanoate increased, although this trend was only significant in the 10:3.3 vs. 10:0.1 (|*t*|=2.91, *P* = 0.022) and 10:3.3 vs. 10:0 (|*t*|=3.13, *P* = 0.012) treatment comparisons (Fig. 6).

**Fig. 7:**
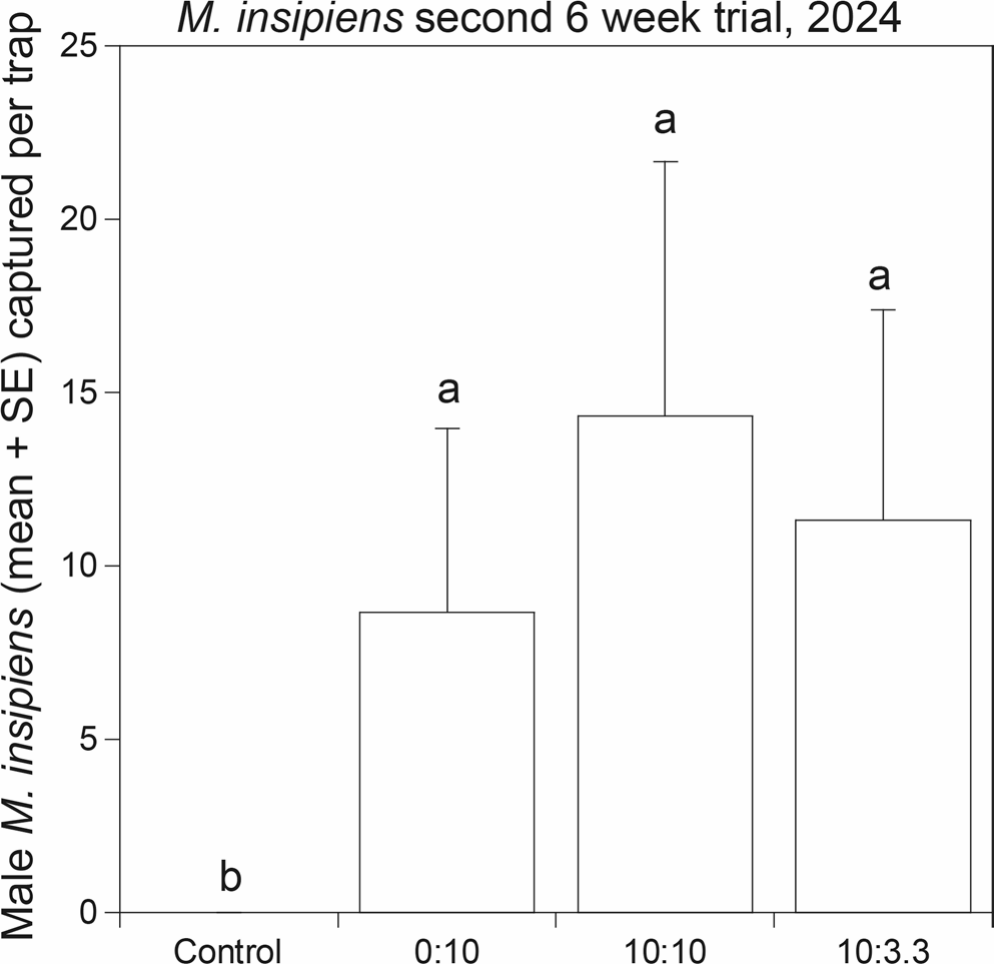
Second 6 wk field bioassay (28 May – 11 July 2024) testing attraction of male *Melanotus insipiens* to the blend of decyl octanoate: decyl butanoate (10:3.3) found in an ovipositor extract (column at the right), against an equal ratio of the two components, or decyl butanoate alone. Columns represent the mean number of male beetles caught per trap for each treatment. Columns surmounted with the same letters are not significantly different (ANOVA, F = 5.33, df = 3,23, *P* = 0.007, *n* = 206, followed by Dunnett’s Test comparing the 10:3.3 blend to each of the other treatments, *P* < 0.05)

**Fig. 8:**
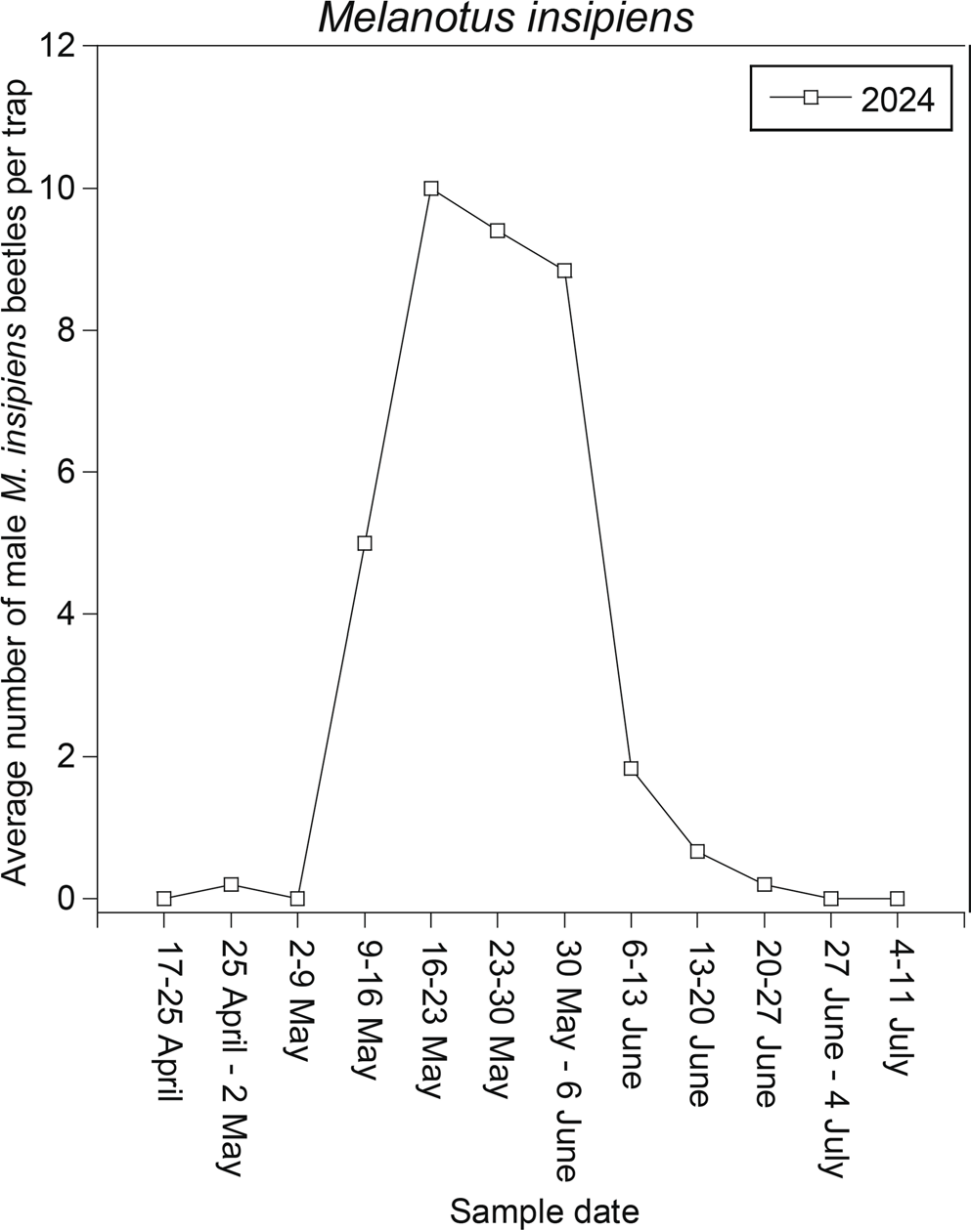
Seasonal phenology of male *Melanotus insipiens* captures with the natural blend of decyl octanoate: decyl butanoate (10:3.3), 2024

Because the results of the first 2024 trial indicated that decyl octanoate alone was not attractive to *M. insipiens*, and because catches appeared to increase with the amount of decyl butanoate present, a second trial tested the 10:3.3 ratio versus a 10:10 ratio, and versus decyl butanoate as a single component. A total of 222 males (206 used in analysis) were captured in traps baited with the treatments, with 0 beetles in controls (*F* = 5.33, df = 3,23, *P* = 0.007) (Fig. 7). All three treatments were significantly greater than the control, but there were no significant differences among the three treatments, i.e., decyl butanoate as a single component was an attractive lure, and addition of decyl octanoate had no effect, either positive or negative (Fig. 7). In 2024, *M. insipiens* beetles were captured with the natural blend of 10:3.3 decyl octanoate: decyl butanoate from 23 April to 5 July (Fig. 8).

## Discussion

In 2023 and 2024 we identified and field-tested putative pheromone components for *M. piceatus* (major component (2*Z*,6*E*)-farnesyl acetate and possible minor component (2*E*,6*E*)-farnesyl acetate) and *M. insipiens* (apparent major component decyl octanoate and minor component decyl butanoate). For *M. piceatus*, we first tested a two-component blend [(2*Z*,6*E*)-farnesyl acetate and (2*E*,6*E*)-farnesyl acetate] against (2*Z*,6*E*)-farnesyl acetate alone. The results suggested that (2*Z*,6*E*)-farnesyl acetate was the primary attractant, and that the addition of (2*E*,6*E*)-farnesyl acetate, if anything, decreased attraction of male *M. piceatus*. Furthermore, only (2*Z*,6*E*)-farnesyl acetate elicited responses from antennae of male beetles in GC-EAD analyses. In combination, these results suggested that the female-produced sex pheromone of *M. piceatus* consists of (2*Z*,6*E*)-farnesyl acetate as a single component.

In similar fashion, for *M. insipiens*, only one of the two compounds identified in ovipositor extracts elicited electrophysiological and behavioral responses, but unexpectedly, it was the minor component decyl butanoate that proved to be active, with decyl octanoate having no discernable effect, either positive or negative. Our first field bioassay tested decyl octanoate as a single component along with several ratios of decyl octanoate to decyl butanoate including the ratio of 10:3.3 that was found in ovipositor extracts. Decyl octanoate alone was inactive, but attraction to lures increases in tandem with increasing amounts of decyl butanoate, suggesting that decyl butanoate might be a crucial synergist. Thus, a second field bioassay tested decyl butanoate alone, along with 10:3.3 and 10:10 ratios of decyl octanoate to decyl butanoate. This revealed that decyl butanoate was not in fact a synergist, but was the sole active component, being as attractive as the other treatments tested (10:3.3 and 10:10). Apparently, decyl octanoate plays no role in attraction, despite being the major component in ovipositor extracts. This unexpected anomaly was confirmed by GC-EAD analyses, whereby antennae of male *M. insipiens* responded strongly to the minor component decyl butanoate in the ovipositor extracts, but not at all to the major component, decyl octanoate. Analogous results were obtained with GC-EAD analyses of a blend of the two synthesized compounds, with the antennae responding only to decyl butanoate. Thus, the available evidence suggests that the female-produced sex pheromone of *M. insipiens* consists of decyl butanoate as a single component.

With the electrophysiological and bioassay data from both species suggesting that their pheromones consist only of single components, the roles of the other components identified in the ovipositor extracts is unclear. They may serve as inhibitory compounds to minimize cross-attraction of congeners that might also use (2*Z*,6*E*)-farnesyl acetate or decyl butanoate respectively as pheromone components. Alternatively, they may simply be artifacts of the way that the extracts were prepared; we analyzed solvent extracts of ovipositors pulled from the abdomens of anesthetized female beetles, because all our attempts to dissect out pheromone glands or to collect the volatiles released by females of *Melanotus* species have been unsuccessful (Williams et al. 2019). Nevertheless, the fact that the two “inactive” components bear strong structural similarities to the actual pheromone components suggests that they might have some role as mediators of conspecific or heterospecific behavior.

This phenomenon of click beetle females producing analogs and homologs of the compounds that constitute their actual pheromones appears to be widespread within the Elateridae. For example, Tolasch et al. (2007) found that pheromone gland extracts of *Elater ferrugineus* L. contained 7-methyloctyl 5-methylhexanoate, 7-methyloctyl octanoate, 7-methyloctyl 7-methyloctanoate, and 7-methyloctyl (*Z*)−4-decenoate, and the full blend of compounds was attractive. However, further study showed that only the latter compound was required to obtain full activity (Svensson et al. 2012). In another genus, *Dalopius marginatus* (L.) gland extracts contained a major component (neryl decanoate) and two homologs, neryl octanoate and neryl dodecanoate, but neryl decanoate alone was sufficient for attraction (Tolasch and Steidle 2022). Similarly, volatiles collected from the crushed abdomens of *Idolus californicus* Desbrochers des Loges contained neryl hexanoate and neryl octanoate, but neryl hexanoate elicited all the activity (Serrano et al. 2022). In the genus *Agriotes* Eschscholtz, both gland extracts and volatiles collected from female *A. sordidus* Illiger contained two major components, geranyl hexanoate and farnesyl hexanoate, but geranyl hexanoate was both necessary and sufficient to obtain full activity, with blends being no more attractive than the single component. In a different genus, for the species *Parallelostethus attenuatus* (Say), volatiles collected from crushed abdomens of females contained hexanoic acid, 1-octanol, 1,8-octanediol, octyl hexanoate, 1,8-octanediol monohexanoate, and 1,8-octanediol dihexanoate, with the latter compound constituting the attractant, and blends with other components actually being less attractive (Millar et al. 2022). Whereas it is unclear why these species are producing what are apparently redundant or even inhibitory compounds, one practical consequence is that their presence, often in comparatively large amounts, complicates the process of trying to sort out what constitutes the active pheromone for each species. Additional examples of elaterid pheromone gland extracts containing complex mixtures of analogs and homologs are listed in Tóth (2013). Given so many cases across multiple genera, additional examples will undoubtedly emerge as pheromone blends are identified for new species.

During our field trials we observed no evidence of cross-attraction of other elaterid species to any of the lures tested, despite numerous elaterid species being present, as evidenced by captures at our field sites using other collecting methods. Thus, our data indicate that (2*Z*,6*E*)-farnesyl acetate and decyl butanoate may be relatively species-specific to *M. piceatus* and *M. insipiens*, respectively. These results suggest that the pheromone components or pheromone blends of *Melanotus* and other click beetle species may be both diverse, but also narrowly tuned, with each species using either relatively unique compounds, or species-specific ratios of compounds that may be shared by congeners.

Sex pheromones or attractants have been identified for four other North American *Melanotus* species. The major sex pheromone component for *M. communis* has been identified as 13-tetradecenyl acetate (Williams et al. 2019), whereas a two-component pheromone blend of 13-tetradecenyl acetate and 13-tetradecenyl hexanoate has been reported for *M. verberans* (Williams et al. 2025). Also, male *M. ignobilis* Melsheimer were strongly attracted to 11-dodecenyl butanoate, suggesting that this compound is a likely pheromone component for this species (Millar et al. 2024). Several decades ago, *Melanotus depressus* (Melsheimer) was reported to be attracted to the tufted apple bud moth (*Platynota idaeusalis* Walker) sex pheromone (2:1 ratio of (*E*)−11-tetradecenyl acetate + (*E*)−11-tetradecen-1-ol) (Brown and Keaster 1983), but it has not yet been confirmed whether *M. depressus* produces one or both of these compounds.

Pheromones have been reported for two other *Melanotus* species, both from Japan. *Melanotus okinawensis* Ôhira uses the single component dodecyl acetate (Iwanaga and Kawamura 2000; Tamaki et al., 1986), whereas a blend of (*E*)−9,11-dodecadienyl butanoate and (*E*)−9,11-dodecadienyl hexanoate is used by *M. sakishimensis* Ôhira (Iwanaga and Kawamura 2000; Tamaki et al. 1990). There are clear similarities between these compounds and those produced by some of their North American congeners. In other *Melanotus* species, related compounds have been identified, although bioassay data demonstrating attraction are lacking. For example, the compounds used by *M. sakishimensis*, (*E*)−9,11-dodecadienyl butanoate and (*E*)−9,11-dodecadienyl hexanoate, have also been identified in extracts of *M. tamsuyensis* Bates (Yen and Chen 1998). The European species *M. punctolineatus* (Pelerin) may use a tetradecenyl butyrate as a pheromone, whereas *M. rufipes* (Herbst) may use a tetradecadienyl butyrate (Tolasch et al. 2007). Finally, pheromone glands of the west-Eurasian *M. fusciceps* (Gyllenhal) and the Holarctic *M. castanipes* (Paykull) contained mixtures of > 22 compounds, which included esters of 12-carbon and 14-carbon saturated and unsaturated alcohols, although the exact structures were not identified (Yatsynin et al. 1996). In sum, the available data indicates that there is substantial conservation of general structural types in pheromone components within the *Melanotus* genus, but it is also clear that pheromones are likely to be species-specific because of the demonstrated lack of attraction of congeners in the field trials described here and in the other studies described above.

To our knowledge, the (2*Z*,6*E*)-farnesyl acetate produced by *M. piceatus* has not been reported from any other click beetle species, but (2*E*,6*E*)-farnesyl acetate) has been found in several *Agriotes* species (El-Sayed 2025). Furthermore, the homolog (2*Z*,6*E*)-farnesyl octanoate has recently been reported as a sex pheromone of *Dalopius maritimus* Brown, and present in extracts of the congener *D. asellus* Brown (van Herk et al. 2025). Similarly, the two *M. insipiens* compounds decyl octanoate and decyl butanoate have not been previously reported from any click beetles, although they have been found in extracts of other insect taxa (El-Sayed 2025). Seasonal activity patterns likely also play a role in reproductive isolation between sympatric congeners (e.g., *M. communis* vs. *M. verberans*). Whatever the case, it is clear that mechanisms for maintaining reproductive isolation are crucial, given the > 1,000 described species in North America.

Insect sex pheromones have been integral components of pest management for more than half a century. More recently, the pheromones of click beetles have been utilized in studies aimed at a better understanding of click beetle biology with emphasis on improving control of pest species (Arakaki et al. 2008a, b; Furlan et al. 2021; Pellegrino et al. 2021; Vernon and van Herk 2022; Rashed and van Herk 2024; Hanks et al. 2025; Schoeppner et al. 2025). Conversely, pheromones have emerged as essential tools in conservation efforts targeting endangered elaterids in natural systems (Tolasch et al. 2007; Svensson et al. 2012; Larsson 2016). Improved knowledge of the role of semiochemical communication in click beetles will contribute to our understanding of their biology, and their roles in natural and managed systems.

## Data Availability

With reasonable request, all data supporting the findings of this study are available from the authors.
